# Supplementary material for: High contiguity Arabidopsis thaliana genome assembly with a single nanopore flow cell
Source: Nat Commun. 2018 Feb 7;9:541. doi: 10.1038/s41467-018-03016-2 (PMC5803254; doi:10.1038/s41467-018-03016-2)
Supplement: Supplementary file 1 — Supplementary Information [file 41467_2018_3016_MOESM1_ESM.pdf]

Supplementary Note

Pacific Biosciences genome sequencing and assembly

20 g young leaf material were frozen in liquid nitrogen and ground to fine powder using mortar and pestle. The powder was directly transferred into lysis buffer and DNA was extracted with a Qiagen genomic DNA extraction kit (Qiagen, Hamburg, Germany) in combination with Qiagen genomic tip columns (500/G; Qiagen, Hamburg, Germany) according to the manufacturer's protocol. DNA quality and quantity was determined with a NanoDrop ND 1000 spectrometer (PeqLab, Erlangen, Germany), a Qubit 2.0 Fluorometer (Thermo Fisher Scientific, Waltham, USA) and by pulse field gel electrophoresis. A total of 10 µg genomic DNA was sheared to a target fragment size of 50 kb using a Megaruptor™ 2 device (Diagenode, Denville, USA). A 30 kb template library was prepared using the BluePippin™ size-selection system according to the manufacturer's protocol (P/N 101-024-600-02, Pacific Biosciences, California, USA). The final library was sequenced on a Pacific Biosciences Sequel instrument following the Magbead loading protocol. A single SMRT cell resulted in 7.08 Gb of raw sequencing data (Supplementary Fig. 1). Raw reads were assembled with Falcon (commit b22b63d, https://github.com/fbemm/onefc-oneasm/falcon.config) and polished with arrow (version 2.2.1, default parameters, PBfal Round 1) as well as pilon (version 1.22, default parameters, PBfal Round 4). The final assembly had a total length of 119 Mb (N50: 11 Mb) spread across 78 contigs (Table 1). Comparisons to optical maps determined the PBfal assemblies to be free of chimeric misjoins, and to harbor one artificial expanded (7,878 bp) and four collapsed (8.5 - 73 kb) assembly regions.

Supplementary Figures

Supplemental Fig. 1. Comparison of read length and GC content of (A) the Oxford Nanopore (ONT) and (B) PacBio (PB) Sequel raw reads. Lower and upper hinges correspond to the 25th and the 75th percentile. Whiskers are extended 1.5 * IQR (IQR is the interquartile range between the 25th and the 75th percentile) from the smallest and highest hinge.

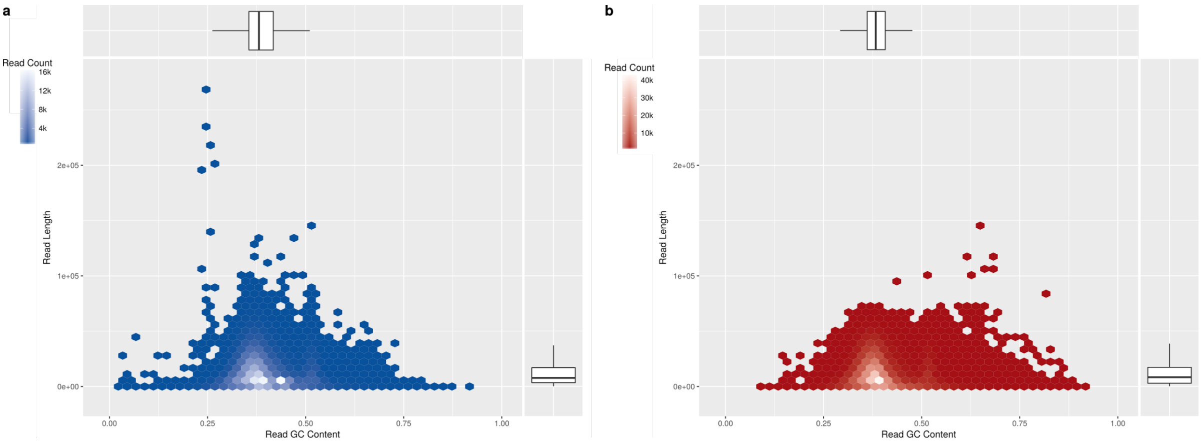

Supplemental Fig. 2. Structural variants between TAIR10 (Col-0) and assembled genomes. (A) ONTmin (Round 4) compared against TAIR10 (Col-0). (B) PBfal (Round 4) compared against TAIR10 (Col-0). Assembly types: Oxford, ONT; PacBio Sequel, PB; Miniasm, min; Falcon, fal. Polishing rounds PBfal: 0 = raw assembly; 1, arrow 1x; 4, pilon 1x.

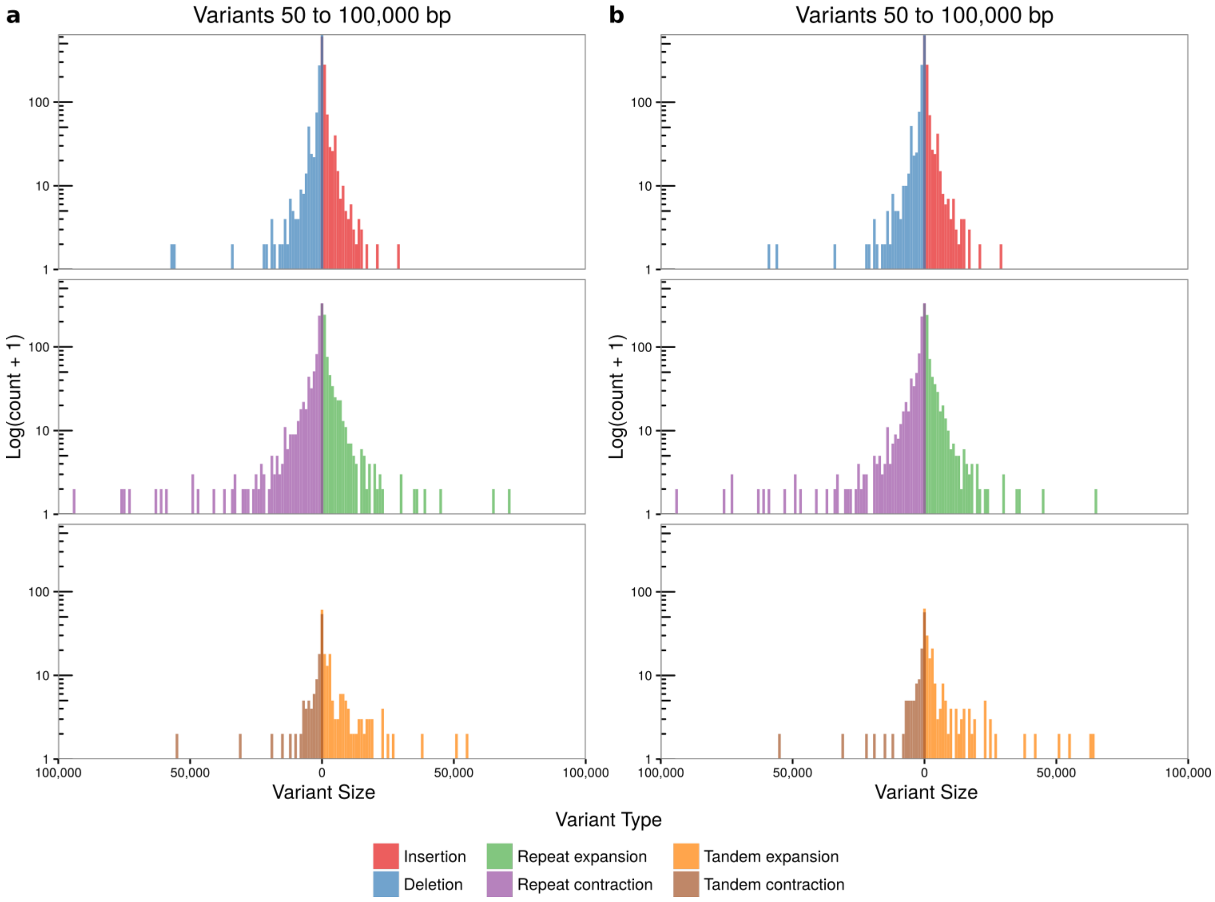

Supplemental Fig. 3. Comparison of heterozygous SNPs in the Oxford Nanopore (ONT), the PacBio (PB) and the TAIR10 reference assembly. Assembly types: Canu, can; Miniasm, min; Falcon, fal; reference genome, TAIR10. Polishing rounds ONTcan and ONTmin: 0 = raw assembly; 1, racon 1x; 2, racon 2x; 3, racon 3x; 4, pilon 1x. Polishing rounds PBfal: 0 = raw assembly; 1, arrow 1x; 4, pilon 1x.

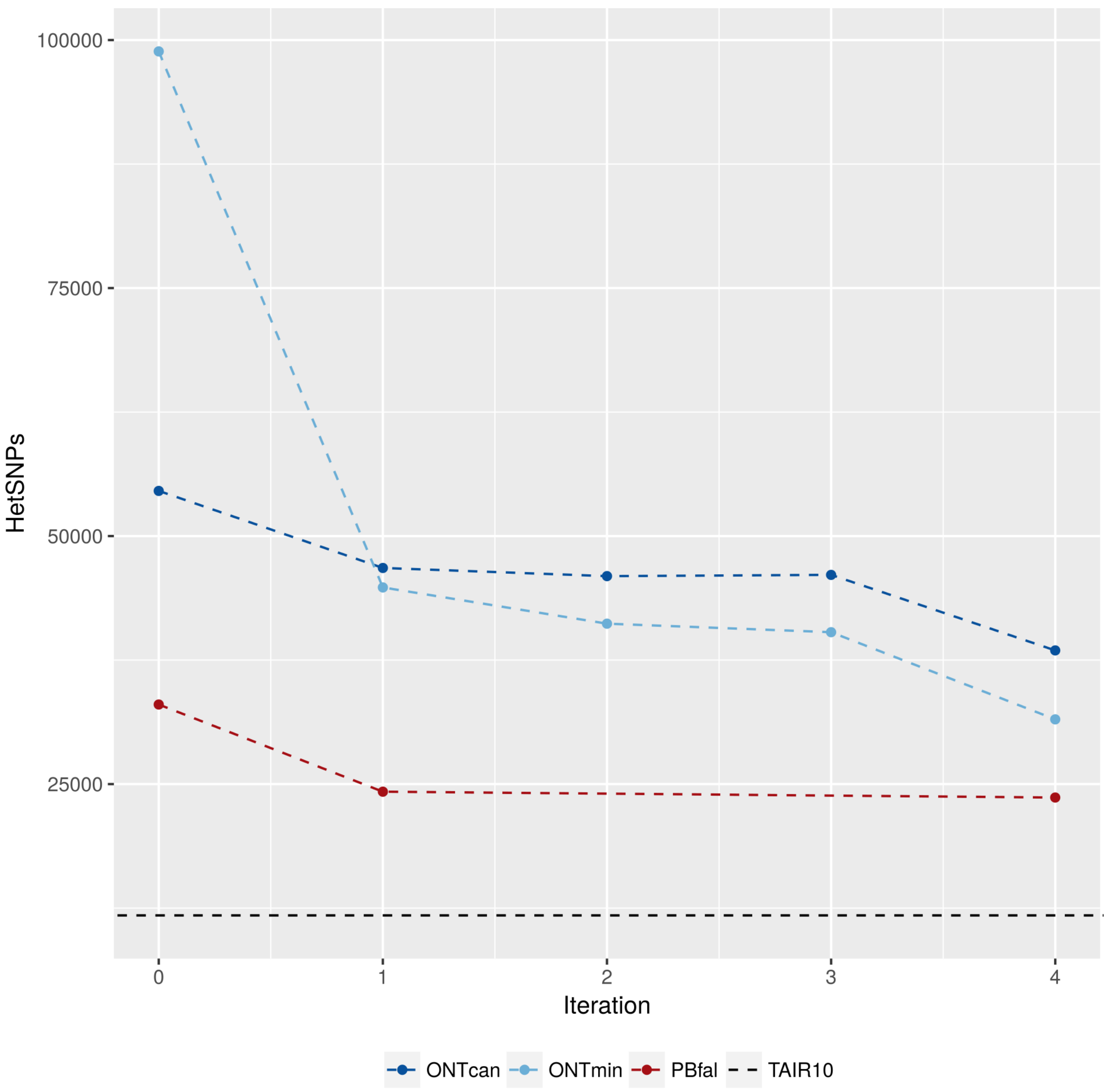

Supplemental Tables

Supplemental Table 1. Bionano Genomics (BNG) Irys mapping statistics to analyze genome assembly integrity. Assembly types: Canu, can; Miniasm, min; Falcon, fal. Polishing rounds ONTcan and ONTmin: 0 = raw assembly; 1, racon 1x; 2, racon 2x; 3, racon 3x; 4, pilon 1x. Polishing rounds PBfal: 0 = raw assembly; 1, arrow 1x; 4, pilon 1x. FP, false positive; FN false negative; # number; Expansion (Exp.) artificial expansion/duplication; Contraction (Con.), artificial contraction/deletion; Cmap, Bionano optical assembled map;

Name	Round (#)	FP Rate (/100kb)	FN Rate (/100kb)	Exp. (#)	Exp. (bp)	Con. (#)	Con. (bp)	Cmap (#)	Contigs covered (#)	Contigs covered (bp)
ONTcan	0	0.33	0.12	4	108,649	4	256,855	139	37	107,211,166
ONTcan	1	0.15	0.08	0	0	5	221,206	175	53	113,172,892
ONTcan	2	0.13	0.07	0	0	4	128,339	194	54	113,378,731
ONTcan	3	0.13	0.07	0	0	4	213,646	164	53	113,297,084
ONTcan	4	0.01	0.04	0	0	4	128,258	167	57	115,815,769
ONTmin	0	-	-	-	-	-	-	1	1	-
ONTmin	1	0.33	0.08	1	17,592	5	176,008	143	30	115,869,843
ONTmin	2	0.22	0.07	1	17,724	6	227,865	143	31	116,269,738
ONTmin	3	0.22	0.07	1	17,735	5	200,697	143	31	116,332,567
ONTmin	4	0.02	0.04	3	32,369	6	244,011	143	31	118,386,082
PBfal	0	0.05	0.32	1	7,465	4	136,303	143	41	118,317,899
PBfal	1	0.01	0.04	1	7,867	4	135,647	144	41	118,721,706
PBfal	4	0.01	0.04	1	7,878	4	135,696	144	41	118,721,706

Supplemental Table 2. Gene completeness statistics for all assemblies according to QUAST metrics. Assembly types: Canu, can; Miniasm, min; Falcon, fal. Polishing rounds ONTcan and ONTmin: 0 = raw assembly; 1, racon 1x; 2, racon 2x; 3, racon 3x; 4, pilon 1x. Polishing rounds PBfal: 0 = raw assembly; 1, arrow 1x; 4, pilon 1x.

Name	Polishing Round (#)	Complete Genes (#)	Partial Genes (#)	Missing Genes (#)
ONTcan	0	25,127	1,979	6,235
ONTcan	1	29,211	1,572	2,558
ONTcan	2	29,376	1,501	2,464
ONTcan	3	29,405	1,498	2,438
ONTcan	4	30,036	1,747	1,558
ONTmin	0	0	2	33,339
ONTmin	1	29,037	1,477	2,827
ONTmin	2	29,408	1,496	2,437
ONTmin	3	29,459	1,515	2,367
ONTmin	4	30,064	1,738	1,539
PBfal	0	29,857	1,672	1,812
PBfal	1	29,873	1,734	1,734
PBfal	4	29,869	1,746	1,726

Supplemental Table 3. Structural variants between TAIR10 (Col-0) and assembled genomes. Assembly types: Oxford, ONT; PacBio Sequel, PB; Miniasm, min; Falcon, fal. Polishing rounds PBfal: 0 = raw assembly; 1, arrow 1x; 4, pilon 1x.

Type	ONTmin_IT4 (#)	ONTmin_IT4 (bp)	PBfal_IT4 (#)	PBfal_IT4 (bp)
Insertions	1,101	1,345,360	1,121	1,379,584
Deletions	1,144	1,617,994	1,165	1,699,713
Tandem Expansions	153	765,310	181	1,020,786
Tandem Contractions	104	282,829	114	313,104
Repeat Expansions	858	2,210,542	848	2,053,061
Repeat Contractions	920	3,293,154	912	3,255,662
